# Analyzing the 20-year declining trend of hospital length-of-stay in European countries with different healthcare systems and reimbursement models

**DOI:** 10.1007/s10754-024-09369-0

**Published:** 2024-03-21

**Authors:** Davide Golinelli, Francesco Sanmarchi, Fabrizio Toscano, Andrea Bucci, Nicola Nante

**Affiliations:** 1 Department of Biomedical and Neuromotor Sciences, Alma Mater Studiorum - University of Bologna, Bologna, Italy; 2 Department of Life Sciences, Health and Healthcare Professions, Link Campus University, Rome, Italy; 3 Department of Medicine, Montefiore Medical Center, Bronx, NY USA; 4 Department of Economics and Law, University of Macerata, Macerata, Italy; 5 Department of Molecular and Developmental Medicine, University of Siena, Siena, Italy

**Keywords:** Length of stay, Healthcare systems, Utilization of services, Comparative health systems, Healthcare model, I18

## Abstract

The study aims to investigate the last 20-year (2000–2019) of hospital length of stay (LOS) trends and their association with different healthcare systems (HS) among 25 European countries. A panel dataset was created using secondary data from Eurostat and Global Burden of Disease study databases, with dependent and control variables aggregated at the national level over a period of 20 years. A time trend analysis was conducted using a weighted least squares model for panel data to investigate the association between LOS, HS models [National Health Service (NHS), National Health Insurance, Social Health Insurance (SHI), and Etatist Social Health Insurance], healthcare reimbursement schemes [Prospective Global Budget (PGB), Diagnosis Related Groups (DRG), and Procedure Service Payment (PSP)], and control variables. The study showed a reduction of average LOS from 9.20 days in 2000 to 7.24 in 2019. SHI was associated with a lower LOS compared to NHS (b = − 0.6327, *p* < 0.05). Both DRG (b = 1.2399, *p* < 0.05) and PSP (b = 1.1677, *p* < 0.05) reimbursement models were positively associated with LOS compared to PGB. Our results confirmed the downward trend of LOS in the last 20 years, its multifactorial nature, and the influence of the SHI model of HS. This could be due to the financial incentives present in fee-for-service payment models and the role of competition in creating a market for healthcare services. These results offer insight into the factors influencing healthcare utilization and can inform the design of more effective, efficient, and sustainable HS.

**Supplementary Information:**

The online version contains supplementary material available at 10.1007/s10754-024-09369-0.

## Introduction

The landscape of Healthcare Systems (HS) around the globe has witnessed significant shifts, reforms, and advancements in recent years (Institute of Medicine, 1988; Marjoua & Bozic, 2012; Baumann, 2021). The driving forces behind these transformations have been the twin principles of patients’ safety and HS sustainability (Kruk et al., 2018; Zurynski et al., 2022). Existing literature suggests that both principles can be distilled into one key indicator—the average length of hospital stay (LOS) (Buttigieg et al., 2018; Chletsos & Saiti, 2019; Siddique et al., 2021; Thomas et al., 1997). This indicator is often utilized to gauge the efficiency and performance of HS (Baek et al., 2018). LOS can be defined as the total number of days spent by patients in inpatient settings during a year, divided by the number of admissions or discharges (Baek et al., 2018).

While a recent literature review conducted by the U.S. Agency for Healthcare Research and Quality did not yield any clear-cut organizational interventions that could reduce LOS (Siddique et al., 2021), it is clear that LOS is influenced by various endogenous and exogenous variables (Chletsos & Saiti, 2019; Thomas et al., 1997). Among the endogenous factors, we can count the health professionals’ intent to minimize LOS for patients for safety and risk management reasons (Baek et al., 2018). In fact, longer hospital stays may result in unfavorable clinical outcomes (e.g., hospital-acquired infections) and should be evaluated in conjunction with other outcome measures (e.g., disease-specific mortality rates within 30 days of hospitalization) (Marfil-Garza et al., 2018; OECD Data, 2022).

Delays in hospital discharge can also be attributed to unwarranted waiting times, inadequate organization of care, and challenges related to discharge planning (GBD 2019 Antimicrobial Resistance Collaborators, 2022a, 2022b; Gruneir et al., 2016; Ragavan et al., 2017; Tipton et al., 2021). Empirical evidence has demonstrated that around 10−20% of hospital admissions can be managed safely as outpatients (Cisse et al., 2016). These avoidable admissions usually involve patients who are likely to have a shorter LOS due to lower acuity, fewer comorbid conditions, and better social support (Wadhera et al., 2019). On the contrary, there have been several exogenous influences that have precipitated a coerced reduction of the average duration of hospital stays, for reasons pertaining to financial and budgetary equilibrium (Cram, 2019; Daniels et al., 2018; Lynch et al., 2019; OECD, 2019; Ofori-Asenso et al., 2020).

Other than the aforementioned factors, LOS may also be significantly reliant on the nature of healthcare policies and governance models delineated at the regional and national level, and as a result, essentially, on the model of HS. Healthcare systems can be categorized in various ways (World Bank Group, 2017; Universal Health Coverage, 2023; Böhm et al., 2013), but the most critical aspects to be considered are the type of financing, reimbursement, and delivery of services. In 2013, Böhm (Böhm et al., 2013) categorized 30 OECD HS according to a deductively generated typology based on a hierarchy of dimensions (regulation—financing—service provision) and the actors accountable for them (state—societal/insurances—private). Their analysis on the overall structures and financing mechanisms of the HS generated 5 clusters of HS’s models (Table 1): the National Health Service (NHS), National Health Insurance (NHI), Social Health Insurance (SHI), Private System (PS) and Etatist Social Health Insurance (ESH).

**Table 1:** Countries included in the study, divided by type of healthcare system

Country	Healthcare system models	Dimensions			
		Regulation	Financing	Provision	Reimbursement
Denmark	National Health Service and Insurance (NHS)	SA	SA	PA	DRG
Finland		ST	SA	PA	PSP
Greece		ST	SA	PA	DRG
Iceland		ST	ST	ST	PSP
Ireland		ST	SA	PA	DRG
Italy		ST	ST	ST	DRG
Norway		ST	SA	PA	DRG
Portugal		SA	SA	PA	DRG
Spain		ST	ST	PA	DRG
Sweden		ST	SA	PA	DRG
United Kingdom		ST	ST	ST	PGB
Austria	Social Health Insurance (SHI)	ST	SA	PA	PGB
Germany		ST	ST	ST	DRG
Luxembourg		SA	SA	PA	PGB
Slovenia		ST	SA	PA	DRG
Switzerland		ST	ST	ST	PSP
Belgium	Etatist Social Health Insurance (ESH)	ST	SA	PA	DRG
Czechia		ST	ST	ST	PSP
Estonia		ST	SA	PA	PSP
France		ST	SA	PA	DRG
Hungary		ST	ST	ST	PSP
Netherlands		ST	ST	ST	PGB
Poland		SA	SA	PA	DRG
Slovakia		ST	SA	PA	PGB
Turkey		ST	ST	ST	PSP

NHS, National Health Service and Insurance; SHI, Social Health Insurance; ESH, Etatist Social Health Insurance; ST, State; SA, Societal actors; PA, Private actors; PGB, Prospective Global Budget; DRG, Diagnosis Related Groups; PSP, Procedure Service Payment

The NHS model, which is prevalent in Nordic European countries, the UK, and some southern European countries, is characterized by state control over regulation, financing, and service provision. The NHI model combines NHS regulatory structures and tax financing with private service provision. The SHI model, on the other hand, is marked by the dominant role of societal actors in healthcare regulation and financing, with private for-profit providers delivering services. This model is mainly seen within the OECD context in German-speaking European countries such as Austria, Germany, Luxembourg, and Switzerland.

The PS model is characterized by coordination by market actors, private financing sources, and for-profit providers. However, this model has only prevailed in the United States since Switzerland switched to the corporatist SHI model in 1996. The ESHI model, which is the only completely mixed healthcare model that exists, is characterized by a clear hierarchy of the three dimensions: the state is responsible for regulating the system, financing is organized by societal actors, and provision is usually delegated to private hands.

The heterogeneity of HS across the globe has resulted in substantial variability in LOS among different countries. This variability has led to a series of concerted efforts in the past decades to reduce it Morris et al., 2016; Samsky et al., 2019; Grover et al., 2018; Arefian et al., 2019; Sun et al., 2013; Hauck & Zhao, 2011; Tiessen et al., 2013). The latest evidence suggests that HS in high-income countries are taking an active stance to manage the duration of hospital stay, albeit with mixed results (Sun et al., 2013; Hauck & Zhao, 2011; Tiessen et al., 2013; Rosenthal et al., 2003). It is plausible that disparities in the HS models may play a pivotal role in shaping policies governing LOS. Remarkably, no empirical evidence exists to establish whether and how differing models of HS could impact hospital average LOS. It is crucial to study the relationship between HS models and LOS since hospitalization is a fundamental element of healthcare utilization and can significantly influence the overall cost and quality of healthcare.

As such, this study aims to investigate the 20-year trends of hospital LOS and their association with distinct HS and reimbursement models among 25 European countries, while adjusting for several exogenous variables. This area of research holds promise and could offer insights into the nature and determinants of LOS variations across different HS models.

## Methods

### Study design and data

We conducted a 20-year time trend analysis (2000–2019) using a weighted least squares (WLS) model to investigate the average LOS in 25 European countries (Table 1).

Secondary data including average LOS, gross domestic product (GDP) per capita, educational level (EL), share (%) of the population having a long-standing illness or health problem, number of generalist medical practitioners (GMP), and number of hospital beds (HB) were extracted from the Eurostat database (Statistics Eurostat, 2022). Data on healthy life expectancy (HLE) were collected from the Global Burden of Disease results tool (Institute for Health Metrics and Evaluation, 2015). Using these data, we created a panel dataset, where dependent and control variables were aggregated at the national level over a period of 20 years.

Our study adheres to the Reporting of Studies Conducted using Observational Routinely Collected Health Data (RECORD) guidelines (Benchimol et al., 2015) (available in the Supplementary material).

### Dependent variable

Average LOS was used as the dependent variable in our study, defined as the average number of days that patients spend in hospital. LOS is computed by dividing the number of hospital days (or bed days or in-patient days) from the date of admission in an in-patient institution (date of discharge minus date of admission) by the number of discharges (including deaths) during the year (Statistics Eurostat, 2022).

### Healthcare system classification by financing, regulation, and service provision

We included the model of HS as the main variable of interest (Table 1). In this study, we decided to use the classification proposed by Böhm and colleagues (Böhm et al., 2013) that subdivides HS into five large clusters (NHS, NHI, SHI, PS and ESH), based on a hierarchy of dimensions that we also considered in the analysis (regulation—financing—service provision) and actors (state—societal/insurances—private). In our sample, no country adopted the PS system model.

Overall, the Böhm et al. classification is based on the relevant actors in charge of HS regulation, financing, and services’ provision, thereby distinguishing between state, societal (insurances and private non-profit) and private (for-profit) actors (Table 1).

They classified each object of regulation according to the actor responsible for regulating the field, relying on the most recent available WHO HS reviews. For the financing dimension, countries were classified using OECD Health Data for the year 2008 which provides health expenditure by financing agents and differentiates between government (state), social security funds (societal), private insurances, and out-of-pocket expenditure (both private). Their classification of health service provision is based on the service provision index developed by Rothgang et al. (Rothgang et al., 2010) focusing on the main items of expenditure in the HS of developed countries: inpatient care, outpatient care, and pharmaceuticals.

In accordance with these principles, we have also classified HS based on the three main three dimensions that define the systems themselves: financing, service provision, and regulation. Therefore, we classified each country based on the type of HS financing modes (State, Societal actors/insurances, Private actors, and Mixed), HS regulation (State, Societal actors/insurances, Private actors, and Mixed), and service provision/delivery (State, Societal actors/insurances, Private actors, and Mixed).

### Healthcare system classification by reimbursement scheme

One aspect not considered in the classification of Böhm et al. is the method of reimbursement for the provision of health services in the various countries. Therefore, to increase the robustness of our analysis and consider the type of healthcare services reimbursement, we decided to include this variable using the OECD classification (OECD/World Health Organization/Eurostat, 2011), which classifies systems into three main reimbursement schemes for healthcare providers (Table 1): Prospective Global Budget (PGB), Diagnosis Related Groups (DRG), and Procedure Service Payment (PSP).

PGB establishes a fixed budget for healthcare providers on an annual basis based on various factors, including the population size, age, health status, and expected healthcare costs. Under this model, healthcare providers are responsible for managing their budgets, ensuring that they provide the best possible care within their budget’s limits. DRG is a healthcare financing model that assigns a fixed payment for specific medical diagnoses or procedures, rather than on the actual cost of care. DRG incentivizes a more efficient and cost-effective care, leading to reduced healthcare costs. However, it may incentivize healthcare providers to over-treat or perform unnecessary procedures to maximize their reimbursement. PSP pays healthcare providers based on the specific services they provide, rather than on the overall cost of care. Reimbursement is provided for each individual service, such as a diagnostic test, a surgical procedure, or a consultation.

### Other control variables

We also included the following control variables in the panel model. GDP: is defined as the sum of gross value added by all resident producers in the economy plus any product taxes not included in the valuation of output, divided by midyear population (DataBank, 2022). We included GDP in thousand euro per capita in the model to account for countries’ economic trends as it is related to health outcomes (Golinelli et al., 2017, 2018; Sanmarchi et al., 2022). GDP2: health status and economic development usually present a nonlinear relationship, as exemplified by the well-established Preston curve (Preston, 2003). In fact, as the income levels rise, the marginal benefits to health outcomes tend to diminish. For this reason, we have included the quadratic term of GDP per capita into our set of covariates. EL is the share (%) of the population with upper secondary education. The negative relationship between educational level and individual health status has been reported by previous studies (Faresjö et al., 2010; Hahn & Truman, 2015; Zajacova & Lawrence, 2018). CM: Illness is defined as the share (%) of the population having a long-standing illness or health problem (duration of at least six months). GMP is the number of generalist medical practitioners per 100,000 population. HB is defined as the number of available beds in hospitals per 100,000 population and comprises Curative care beds, Rehabilitative care beds, Long-term care beds, and other hospital beds. HLE is defined as the average number of years that a person can expect to live in "full health" (Benchimol et al., 2015; Global Burden of Disease Collaborative Network, 2020).

### Statistical analysis

The association between average LOS and the independent variables was investigated through weighted least squares (WLS) models for panel data. This specification allows to estimate a single set of coefficients across all countries, and to account for heteroskedasticity in the error term by using weights that reflect the variance of the dependent variable within each country. This approach can be useful when there is limited within-country variation and avoids estimating individual fixed effects. Categorical variables that are constant over time, such as the HS, can be included as regressors in the model.

To account for the different dimensions of HS, we specified six different models with common control variables. Model #1 considered the 3 types of HS as classified by Bohm’s et al. (i.e., NHS, NHI, and SHI). Model #2 to #4 considered one of the 3 dimensions on which this classification is based (i.e., regulation, financing, and provision). Model #5 considered the method/scheme of reimbursement for the provision of health services. Model #6 considered both the 3 models of HS and the method of reimbursement and should be considered as the main estimation model.

Additional information on the WLS methodology can be found in the Supplementary Material. In Supplemental Material, we also performed robustness checks for the selection of the covariates, using a general-to-specific approach, excluding one regressor at a time. We also tested for the exlusion of possibile outlier countries.

## Results

### Descriptive statistics

**Fig. 1:**
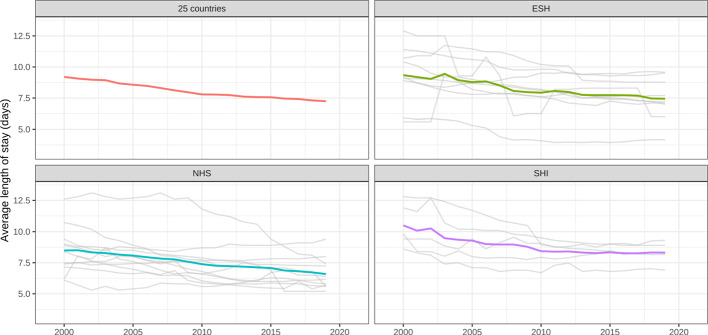
Time trend (2000–2019) of the average length of stay by type of healthcare system. *Notes*. The figure was created using the ggplot2 package (R software). NHS, National Health Service and Insurance; SHI, Social Health Insurance; ESH, Etatist Social Health Insurance

Our results showed a generalized reduction of the average LOS across the 25 countries during the study period (Table 1, 2). The overall average LOS was 9.20 (SD = 2.15) days in 2000 and decreased to 7.24 (SD = 1.51) days in 2019 across the whole set of countries, but the reduction was more marked in countries with NHI HS (9.34 to 7.45), as compared to NHS and SHI (from 8.49 to 6.59 and from 10.51 to 8.31, respectively). Despite starting from different values of LOS, the downward trend was similar for NHS and ESH, while SHI showed a steeper reduction from 2000 to 2010 and then plateaued around 2010 (Fig. 1).

**Table 2:** Average (and standard deviation) of dependent and control variables by model of healthcare system

	2000	2019
Average length of stay (days)		
Overall	9.20 (2.15)	7.24 (1.51)
NHS	8.49 (1.95)	6.59 (1.28)
SHI	10.51 (1.77)	8.31 (0.92)
ESH	9.34 (2.40)	7.45 (1.73)
GDP per capita (thousand €)		
Overall	27.19 (17.04)	34.38 (19.12)
NHS	30.89 (12.67)	38.21 (16.45)
SHI	39.84 (22.76)	48.24 (24.85)
ESH	15.64 (11.94)	22.01 (11.42)
GP per 100,000		
Overall	90.57 (38.86)	102.81 (53.07)
NHS	99.07 (41.61)	111.24 (68.45)
SHI	79.97 (36.30)	104.38 (31.92)
ESH	86.08 (38.92)	91.65 (42.95)
Educational level (%)		
Overall	20.94 (7.04)	30.26 (7.24)
NHS	20.66 (7.53)	32.23 (7.62)
SHI	24.66 (8.62)	32.04 (6.12)
ESH	19.22 (5.33)	26.88 (6.76)
Chronic morbidity (%)		
Overall	32.38 (5.59)	34.69 (7.37)
NHS	29.94 (5.01)	33.31 (9.33)
SHI	35.44 (3.41)	35.40 (6.50)
ESH	33.68 (6.37)	35.99 (5.32)
Hospital beds per 100,000		
Overall	570.45 (187.68)	439.50 (164.81)
NHS	461.73 (121.56)	310.61 (54.99)
SHI	644.88 (219.32)	567.88 (173.30)
ESH	661.97 (184.42)	525.70 (145.55)
Healthy life expectancy (years)		
Overall	66.91 (1.90)	69.99 (1.46)
NHS	67.80 (1.15)	70.66 (0.92)
SHI	67.56 (0.91)	70.74 (0.85)
ESH	65.45 (2.24)	68.77 (1.53)

NHS, National Health Service and Insurance; SHI, Social Health Insurance; ESH, Etatist Social Health Insurance

Regarding the control variables, the overall average GDP per capita (in thousand euros) was 27.19 (SD = 17.04) in 2000 and rose to 34.38 (SD = 19.12) in 2019. Overall, the mean number of hospital beds per 100,000 inhabitants was 570.45 (SD = 187.68) in 2000 and decreased to 439.50 (SD = 164.81) in 2019. The complete description of the variables entailed in the analysis is presented in Table 2.

Average (and standard deviation) of dependent and control variables by the relevant actor in charge of regulation, financing, and services’ provision are available in Supplementary Table S.1.

### Panel model estimation

The panel model estimates are reported in Table 3. In all the models (#1 to #6), GDP (except for Model #1), GMP, the HB, CM, and HLE had a statistically significant positive association with LOS, while EL had a significant negative association with it (except for Models #3 and #4). Moreover, the coefficient associated with the temporal trend is always significantly negative, which confirms the decreasing trend through the observed period of LOS.

**Table 3:** Results of the estimates in the WLS panel model for the period 2000–2019

	(1)	(2)	(3)	(4)	(5)	(6)
GDP	0.0103*	0.0069	0.0053	0.0002	0.0178*	0.0246*
GMP	0.0066*	0.0079*	0.0087*	0.0076*	0.0067*	0.0061*
EL	− 0.0404*	− 0.0360*	− 0.0279*	− 0.0258*	− 0.0389*	− 0.0446*
CM	0.0782*	0.0728*	0.0702*	0.0651*	0.0737*	0.0749*
HB	0.0060*	0.0058*	0.0067*	0.0068*	0.0042*	0.0046*
HLE	0.2160*	0.1935*	0.1601*	0.1933*	0.1681*	0.1643*
Time trend	− 0.0922*	− 0.0914*	− 0.0817*	− 0.0857*	− 0.0951*	− 0.0908*
SHI (vs NHS)	− 0.5077*					− 0.6327*
ESH (vs NHS)	0.0425					0.0092
Regulation SA (vs ST)		− 0.0303				
Financing SA (vs ST)			− 0.4914*			
Provider PA (vs ST)				− 0.6171*		
Reimbursement DRG (vs PGB)					1.2399*	1.2917*
reimbursement PSP (vs PGB)					1.1677*	1.1488*
Log-likelihood	− 717.52	− 717.09	− 716.48	− 714.09	− 713.16	− 711.00
AIC	1455.0	1452.2	1450.9	1446.2	1446.3	1446.0
BIC	1497.6	1490.5	1489.2	1484.5	1488.9	1497.0
${R}^{2}$	0.7575	0.7710	0.8273	0.7946	0.8633	0.8376

*Denotes a coefficient significant at 5%. All the covariates, except for the time trend and the categorical variables NHS, SHI, ESH, ST, SA, PA, PGB, DRG, and PSP, have been lagged once

GDP, Gross Domestic Product per capita; GMP, General medical practitioners per 100,000 population; EL, share (%) of the population with upper secondary education; CM, share (%) of the population having a long-standing illness or health problem (duration of at least six months); HB, hospital beds per 100,000 population; HLE, healthy life expectancy (years); NHS, National Health Service and Insurance; SHI, Social Health Insurance; ESH, Etatist Social Health Insurance; ST, State; SA, Societal actors; PA, Private actors; PGB, Prospective Global Budget; DRG, Diagnosis Related Groups; PSP, Procedure Service Payment

As for estimation model #1, SHI (Countries with Social Health Insurance model) was associated with a lower LOS compared to the reference category (NHS, Countries with National Health Service, and Insurance model) (b = -0.7689, *p* < 0.05).

Models #2 through #4 separately investigated the dimensions on which Bohm’s classification is based, along with the previously listed control variables. Model #2, #3 and #4 showed a significant negative association between the investigated dominion and LOS, when compared with the respective reference category. For instance, model #3 highlighted the negative association between the societal actors/insurances-governed financing scheme (b = -0.6925, *p* < 0.05) and LOS as compared to the State-governed. Model #5 considered the HS reimbursement method/scheme and showed how both DRG (b = 1.0814, *p* < 0.05) and PSP (b = 1.1666, *p* < 0.05) reimbursement models were positively associated with LOS when compared to the PGB reimbursement model.

Model #6 accounts for both the Bohm classification of HS (NHS vs SHI vs ESH) and the reimbursement method/scheme (PGB vs DRG vs PSP). This confirmed the associations discovered in Model #1 and #5, albeit with slightly different coefficients.

All tested models presented a good R-squared ranging from 0.81 to 0.87.

Furthermore, the results of the robustness checks indicate that, while the exclusion of certain covariates does lead to differences, the overall pattern of SHI models being associated with shorter LOS remains consistent. Finally, in the same section, we have also assessed that, excluding two possible outlier countries (i.e., Finland and Turkey) the results remain unchanged.

## Discussion

The present study delved into the temporal trend of average LOS and its correlation with various HS and reimbursement models across 25 European countries. The findings revealed a noteworthy decline in average LOS from 2000 to 2019 across the analyzed sample and a substantial influence of HS models on LOS. The observed trend of decreasing LOS in high-income European countries is comparable to that of other HS, even more accentuated in the U.S. system (Ofori-Asenso, 2020), which, it should be noted, was not encompassed within this analysis. The U.S. HS has been classified (Böhm et al., 2013; Goodney et al., 2003) as predominantly private in nature. Recently, the U.S. HS has shown a decreasing trend in LOS; however, this trend has been more heterogeneous in nature, partially due to the fragmentation of the system itself. For example, Doctoroff and Mukamal (2019) conducted a comprehensive examination of longitudinal trends in LOS within academic medical centers in the U.S. from 2007 to 2016, highlighting two distinct trends. LOS declined steadily from 2007 to 2010. However, beginning in 2011, the mean LOS began to rise steadily and ultimately reached its zenith in 2016. This highlights how medical centers, situated within a private HS model, were able to reduce LOS during the initial years, but then confronted novel forces that sought to increase LOS in subsequent years. A possible explanation resides in the complex mix of fee-for-service and bundled payments that characterize US physicians and hospitals, and the trend inversion observed in the second part of the study period, could be explained by a transition towards value-based programs, such as the Hospital Readmission Reduction Program.

In our analysis, two out of three HS models, namely, NHS and ESH, showed a consistent and uniform downward trend in average LOS, while SHI plateaued around 2012 and did not experience any significant reduction thereafter. The downward trend recorded in the last 20 years shows the efforts made by the European Countries to keep this important indicator under control.

Our study analyzed the impact of the main dimensions (i.e., financing, service delivery, and regulation) which define different HS models, on LOS. Our analyses indicated that financing and service provision were significantly associated with LOS, while the type of regulation was not. Additionally, both DRG and PSP reimbursement schemes were linked with higher LOS compared to PGB.

A key finding of our study is the dominant influence of HS models on LOS. Specifically, SHI models were associated with shorter LOS compared to NHS, after accounting for several endogenous and exogenous factors. One possible explanation for this observation is the need for SHI models to keep their finances under better control in countries with a prominent role of societal actors in healthcare regulation and financing (Barnish et al., 2018; Bekker et al., 2018; Bigdeli et al., 2020; Borrell et al., 2007; Englum et al., 2016; Mainous et al., 2011; Nuti et al., 2016, 2021; Saltman & Duran, 2015; Stewart et al., 2020; Thomas et al., 1997; Toth, 2016). The association between SHI systems and shorter hospital stays could also be attributed to the financial incentives present in payment models. SHI systems have mandatory participation of individuals and employers in a health insurance scheme, with PGB as the main reimbursement scheme (40% of SHI countries).-

This is in contrast to NHS systems, which are primarily funded through general taxation, offer free healthcare to all residents, have providers that operate within publicly owned hospitals, and where DRG remains the most used reimbursement scheme.

Our analysis showed that Diagnosis-Related Group (DRG) and Provider-Specific Payment (PSP) reimbursement schemes were linked to a longer LOS when juxtaposed with the PGB model. The latter, PGB, enables a heightened control over allocated resources, promoting more judicious resource utilization. However, it is crucial to understand that while PGB can drive efficiency, it might also create incentives for healthcare providers to minimize their expenses possibly at the cost of optimal patient care, which can include managing LOS stringently. This happens by creating an economic incentive to minimize the hospitalization duration for patients. Consequently, there is a significant financial interest in discharging patients as quickly as possible, and thus reducing the LOS of patients in the hospital (Doetinchem et al., 2010). On the other hand, DRG can aid providers in predicting and subsequently controlling the cost of care. Meanwhile, PSP, frequently employed in fee-for-service health systems, permits providers to bill for each individual service rendered. A potential downside of PSP is the inadvertent incentive it might create for healthcare providers to increase service provision beyond what is strictly necessary, thereby maximizing their remuneration.

Given this context and the data provided in our study, it is appropriate to infer that the higher likelihood of SHI countries to adopt PGB might be a substantial factor contributing to the observed shorter LOS among this group. Another possible explanation is the role of competition in SHI systems. In many SHI systems, patients can choose their healthcare provider. The level of competition present in SHI systems creates a competitive market, where healthcare providers must compete for patients by delivering high-quality and efficient care. In contrast, NHS systems typically provide less choice to patients regarding healthcare providers. Providers in competitive markets may be more likely to employ cost-saving measures such as reducing hospital stays, to attract patients. Thirdly, the management structure of SHI systems, where providers are typically owned by private companies or quasi-public institutions, creates an incentive for providers to operate efficiently and minimize costs to remain profitable. This contrasts with NHS systems, where the providers are typically government-owned, and thus may have less of an incentive to operate efficiently or minimize costs (Hahn & Truman, 2015; Nuti et al., 2021; Toth, 2016).

Another factor that could contribute to the LOS differences between HS is the level of post-acute care. An abundant supply of post-acute care can be considered a key factor for controlling its length. Patients in countries with well-developed post-acute facilities are far more likely to be discharged to post-acute care than their peers in other countries (Englum et al., 2016). However, post-acute care is expensive (Clarke, 1996) and may offer little economic return for private-based systems. Moreover, if access to post-acute care is restricted through funding cuts, capitation, or other "shared risk models," it is possible that longer LOS may be observed. The persistent challenges faced by Canada with regards to hospitalized patients who become alternate levels of care and the bed-blocking predicament experienced by the United Kingdom serve as a testament to the potential consequences of underfunding post-acute care (Avele et al., 2019; Saenger et al., 2022; Quercioli et al., 2018; Nante et al., 2000).

In summary, it is our hypothesis that the variance in average LOS between SHI and NHS models could be partially explained by the different governance capacities determined by the structural differences of HS, and the varying levels of political and financial accountability. SHI models seem to be associated with shorter LOS when compared to NHS ones. This could be due to the financial incentives that are inherent in fee-for-service payment models, the role of competition in creating a market for healthcare services, and the management structures of SHI systems.

Our discovery on the potential ramifications of hospital LOS may have significant consequences for HS models outside the scope of our research, especially the United States' mainly privatized HS. As we have previously noted, private healthcare entities in the U.S. are primarily motivated by profit, which may lead to diverse LOS for patients. Specifically, these hospitals may either extend the LOS, seeking increased revenue (Tynkkynen & Vrangbæk, 2018), or hasten bed turnover, aiming to generate even greater profits. This variance in LOS can result in escalated healthcare expenses, a critical issue for the U.S. system and its citizens (Toth, 2016, Cram et al., 2019). In line with these concerns, the U.S. HS could adopt alternate reimbursement programs, such as pay-for-performance (PGB), and value-based care to curb the duration of hospital stays. Furthermore, our research recommends the implementation of social health insurance schemes as a potential strategy to reduce hospital stays, thus suggesting a role for universal healthcare coverage.

Apart from the type of HS, other factors also influenced LOS in our sample. The proportion of people over the age of 65 affected by at least one chronic condition was found to be related to higher LOS confirming previous evidence (Ayele et al., 2019; Costa & Hirdes, 2010; Hawkes, 2016; Quercioli et al., 2018; Saenger et al., 2022). Furthermore, higher life expectancy was associated with higher LOS. This is not surprising, given that elderly people have a greater likelihood of multimorbidity, which means they require more resources to address their health needs.

Our analysis revealed a significant positive correlation between the number of hospital beds and the countries’ LOS. This finding could be explained by the constraints imposed by a lack of resources, and it reinforces the hypothesis that LOS is highly influenced by economic and financial factors. Efficiency becomes imperative when hospitals are short on resources and beds (Clarke, 1996; Cram, 2019; Englum et al., 2016; Walsh et al., 2022). To treat the same number of patients with similar characteristics, a hospital with fewer beds must decrease the LOS, while a hospital with more resources can afford a longer LOS. A relative abundance of resources (i.e., hospital beds) could lead to higher LOS as physicians are not pressured to discharge their patients rapidly to make room for new ones (Walsh et al., 2022).

Furthermore, a higher number of GMPs was linked to a higher LOS. This relationship may appear counterintuitive, as current evidence suggests that investing in primary health care should provide better health outcomes for the assisted population and reduce the number of hospitalizations (Sanmarchi et al., 2022; Golinelli et al., 2017; Sepehri et al., 2006; Mawajdeh et al., 1997; Regenbogen et al., 2019). However, the availability of effective primary healthcare providers sought to enhance the appropriateness of hospital admissions by treating patients with diminished care needs outside the hospital setting and reserving hospital admission for individuals who genuinely require it (Englum et al., 2016; Clarke, 1996; Sepehri et al., 2006; Mawajdeh et al., 1997; Regenbogen et al., 2019). This may lead to an elongation of LOS owing to the heightened clinical complexity of hospitalized patients.

### Study limitations

Our study had several limitations. Some countries included in this study possess a partially nascent HS, making them potentially unable to effectively regulate certain aspects of healthcare. The association between SHI systems and shorter hospital stays is not definitive and may be influenced by other factors. The quality of healthcare within a given country is one such factor that may play a role in determining the LOS. Furthermore, the study designs utilized in this research could lead to selection bias and confounding. Another limitation of this study is the assumption that HS models remained static between 2000 and 2019. We have confirmed that no apparent structural changes occurred during this timeframe. Additionally, it is important to note that this study solely employed ecological-level data, which runs the risk of the "ecological fallacy." However, because this study only uses the country itself as the unit of analysis and does not generalize about individuals or specific population groups within each region, it does not risk an ecological fallacy (Schwartz, 1994). Lastly, our research could not address the core question of how population health outcomes (e.g., 30-day mortality and rehospitalization), are related to LOS.

## Conclusions

The study’s aim was twofold. Firstly, we sought to explore the trend in average LOS across 25 European countries, and secondly, to investigate the impact of HS models on this critical indicator. Our goal was to cast light on the influence of HS models on healthcare utilization and identify areas where enhancements could be made to promote more effective and cost-efficient care. The findings revealed a downward trend in LOS over the last two decades, underscoring the multifaceted nature of hospital LOS and the significant impact of HS models.

These results have implications for policymakers and healthcare providers, as they offer insight into the factors influencing healthcare utilization and can inform the design of more effective, efficient, and sustainable HS in the long term. As healthcare professionals and stakeholders grapple with questions surrounding LOS reduction, policymakers at the global level must address key concerns. How far can LOS reduction go, and to what extent does scientific evidence support these decisions? A decrease in LOS, bolstered by enhanced post-acute care, primary care, and out-of-hospital care, could potentially address the changing healthcare requirements of increasingly elderly populations beset by multiple chronic pathologies, regardless of HS type. Nevertheless, to monitor the LOS trend, linking LOS data to other patient-centered outcomes, patient safety, and quality metrics is crucial. Doing so is vital for constructing robust governance systems based on data and accountability, and less susceptible to political pressures, to ensure their balanced and sustained improvement.

## Supplementary Information

Below is the link to the electronic supplementary material.

Supplementary file1 (DOCX 86 kb)

## Data Availability

The datasets generated during and/or analyzed during the current study are available from the corresponding author on reasonable request.
