# Supplementary material for: Analyzing the 20-year declining trend of hospital length-of-stay in European countries with different healthcare systems and reimbursement models
Source: Int J Health Econ Manag. 2024 Mar 21;24(3):375–92. doi: 10.1007/s10754-024-09369-0 (PMC11457716; doi:10.1007/s10754-024-09369-0)
Supplement: Supplementary file 1 — Supplementary file1 (DOCX 86 kb) [file 10754_2024_9369_MOESM1_ESM.docx]

**Supplemental Material**

Weighted Least Squares model

Let $y_{i,t}$ be the observed length of hospital stay (LOS) at time $t$ for the country $i$, for $i=1,\ldots,25$ and $t=1,\ldots,T$ (with $T=20$), and $x_{i,t-1}$ be the $k$-dimensional vector of lagged independent variables, the model can be written as follows

$$y_{i,t}= x_{i,t-1}^{'}\beta+\gamma t+\theta z_{i}+ \varepsilon_{i,t}$$

where $\beta$ is a $k\times1$ vector of coefficients, $\gamma$ is a temporal trend effect which helps us understanding possible deterministic changes in the dependent variable due to the temporal evolution and can somehow approximate the technological evolution as well as the definition of new protocols, $z_{i}$ is the time-invariant variable identifying the HS either in terms of financing, regulation and service provision or in terms of reimbursement scheme, $\varepsilon_{i,t}$is the normally distributed error term with mean zero and variance $\sigma_{i}^{2}W_{it}$, where $W_{it}$ is an $n\times n$ diagonal matrix of weights that reflect the variance of the dependent variable within each unit. The use of lagged independent variables has a twofold purpose: on the one hand, it somehow avoids the correlation between the error term and the covariates; on the other hand, it permits to investigate a short-term effect and leaves the possibility of producing predictions from our model.

***Table S1****. Average (and standard deviation) of dependent and control variables by the relevant actor in charge of regulation, financing, and services’ provision.*

| **Regulation** | **2000** | | | **2019** | | |
| --- | --- | --- | --- | --- | --- | --- |
|  | **Overall, N = 25** | **ST** | **SA** | **Overall, N = 25** | **ST** | **SA** |
| **LOS** | 9.20±2.15 | 8.86±2.10 | 10.98±1.63 | 7.24±1.51 | 6.97±1.48 | 8.67±0.54 |
| **LOS** | 27,194.00±17,042.57 | 23,553.33±14,132.30 | 46,307.50±20,303.54 | 34,382.40±19,115.74 | 30,434.76±16,120.58 | 55,107.50±22,561.27 |
| **GDP** | 90.57±38.86 | 92.19±39.19 | 82.08±41.56 | 102.81±53.07 | 100.77±56.86 | 113.55±28.25 |
| **GMP** | 20.94±7.04 | 20.37±6.54 | 23.98±9.80 | 30.26±7.24 | 29.71±7.39 | 33.17±6.43 |
| **EL** | 32.38±5.59 | 32.08±6.05 | 34.00±1.28 | 34.69±7.37 | 34.58±7.53 | 35.30±7.50 |
| **CM** | 570.45±187.68 | 551.29±175.85 | 671.04±244.07 | 439.50±164.81 | 409.11±146.62 | 599.04±183.21 |
| **HB** | 66.91±1.90 | 66.72±2.01 | 67.90±0.59 | 69.99±1.46 | 69.83±1.50 | 70.84±0.95 |
|  |  |  |  |  |  |  |
| **Financing** | **2000** | | | **2019** | | |
|  | **Overall, N = 25** | **ST** | **SA** | **Overall, N = 25** | **ST** | **SA** |
| **LOS** | 9.20±2.15 | 8.60±2.02 | 9.60±2.20 | 7.24±1.51 | 6.64±1.34 | 7.65±1.51 |
| **GDP** | 27,194.00±17,042.57 | 30,657.00±13,333.39 | 24,885.33±19,218.48 | 34,382.40±19,115.74 | 36,070.00±15,650.82 | 33,257.33±21,576.90 |
| **GMP** | 90.57±38.86 | 97.03±43.27 | 86.27±36.54 | 102.81±53.07 | 104.05±67.62 | 101.99±43.44 |
| **EL** | 20.94±7.04 | 20.12±7.71 | 21.49±6.77 | 30.26±7.24 | 31.58±7.71 | 29.39±7.04 |
| **CM** | 32.38±5.59 | 30.43±5.00 | 33.69±5.75 | 34.69±7.37 | 33.88±9.63 | 35.23±5.73 |
| **HB** | 570.45±187.68 | 446.58±116.68 | 653.02±182.66 | 439.50±164.81 | 312.87±57.43 | 523.92±159.23 |
| **HLE** | 66.91±1.90 | 67.92±1.13 | 66.23±2.03 | 69.99±1.46 | 70.69±0.96 | 69.53±1.58 |
|  |  |  |  |  |  |  |
| **Provision** | **2000** | | | **2019** | | |
|  | **Overall, N = 25** | **ST** | **PA** | **Overall, N = 25** | **ST** | **PA** |
| **LOS** | 9.20±2.15 | 8.62±2.15 | 9.53±2.15 | 7.24±1.51 | 6.79±1.32 | 7.50±1.58 |
| **GDP** | 27,194.00±17,042.57 | 32,126.67±13,255.28 | 24,419.38±18,660.13 | 34,382.40±19,115.74 | 38,104.44±15,132.85 | 32,288.75±21,202.25 |
| **GMP** | 90.57±38.86 | 98.27±45.71 | 86.24±35.30 | 102.81±53.07 | 110.70±68.16 | 98.38±44.39 |
| **EL** | 20.94±7.04 | 20.57±8.03 | 21.16±6.68 | 30.26±7.24 | 32.21±7.90 | 29.17±6.86 |
| **CM** | 32.38±5.59 | 30.56±5.28 | 33.41±5.66 | 34.69±7.37 | 35.01±9.49 | 34.51±6.24 |
| **HB** | 570.45±187.68 | 443.25±123.25 | 642.00±181.90 | 439.50±164.81 | 301.19±46.63 | 517.30±156.09 |
| **HLE** | 66.91±1.90 | 67.91±1.19 | 66.34±2.01 | 69.99±1.46 | 70.77±0.98 | 69.56±1.53 |

*GDP: Gross Domestic Product per capita. GMP: General medical practitioners per 100,000 population. EL: share (%) of the population with upper secondary education. CM: share (%) of the population having a long-standing illness or health problem (duration of at least six months). HB: hospital beds per 100,000 population. HLE: healthy life expectancy (years). NHS: National Health Service and Insurance. SHI: Social Health Insurance. ESH: Etatist Social Health Insurance. ST: State. SA: Societal actors. PA: Private actors.*

*Table S2-S8. Robustness checks*

*Table S2. Results of the estimates in the WLS panel model for the period 2000-2019 excluding the GPD and its square.*

|  | *(1)* |  | *(2)* |  | *(3)* |  | *(4)* |  | *(5)* |  | *(6)* |  |
| --- | --- | --- | --- | --- | --- | --- | --- | --- | --- | --- | --- | --- |
| *GMP* | *0.0071* | *** | *0.0081* | *** | *0.0071* | *** | *0.0075* | *** | *0.0069* | *** | *0.0070* | *** |
| *EL* | *-0.0384* | *** | *-0.0311* | *** | *-0.0321* | *** | *-0.0325* | *** | *-0.0245* | *** | *-0.0302* | *** |
| *CM* | *0.0754* | *** | *0.0686* | *** | *0.0728* | *** | *0.0679* | *** | *0.0662* | *** | *0.0635* | *** |
| *HB* | *0.0054* | *** | *0.0057* | *** | *0.0059* | *** | *0.0054* | *** | *0.0044* | *** | *0.0040* | *** |
| *HLE* | *0.2872* | *** | *0.2205* | *** | *0.2523* | *** | *0.2478* | *** | *0.2489* | *** | *0.2742* | *** |
| *Time trend* | *-0.1149* | *** | *-0.1005* | *** | *-0.1049* | *** | *-0.1087* | *** | *-0.1129* | *** | *-0.1190* | *** |
| *SHI (vs NHS)* | *-0.7165* | *** |  |  |  |  |  |  |  |  | *-0.3491* | *** |
| *ESH (vs NHS)* | *0.2297* |  |  |  |  |  |  |  |  |  | *0.3256* | *** |
| *Regulation SA (vs ST)* |  |  | *0.0622* |  |  |  |  |  |  |  |  |  |
| *Financing SA (vs ST)* |  |  |  |  | *-0.5060* | *** |  |  |  |  |  |  |
| *Provider PA (vs ST)* |  |  |  |  |  |  | *0.2205* |  |  |  |  |  |
| *reimbursement DRG (vs PGB)* |  |  |  |  |  |  |  |  | *1.1637* | *** | *1.1553* | *** |
| *reimbursement PSP (vs PGB)* |  |  |  |  |  |  |  |  | *1.1858* | *** | *1.0652* | *** |
| *Log-likelihood* | *-715.00* |  | *-712.77* |  | *-710.29* |  | *-713.33* |  | *-705.21* |  | *-710.37* |  |
| *AIC* | *1448.0* |  | *1441.5* |  | *1436.6* |  | *1442.7* |  | *1428.4* |  | *1442.7* |  |
| *BIC* | *1463.0* |  | *1454.9* |  | *1449.9* |  | *1456.0* |  | *1443.4* |  | *1461.1* |  |
| $R^{2}$ | *0.7484* |  | *0.7602* |  | *0.7577* |  | *0.7530* |  | *0.8158* |  | *0.7838* |  |

*Notes. * denotes a coefficient significant at 5%. All the covariates, except for the time trend and the categorical variables NHS, SHI, ESH, ST, SA, PA, PGB, DRG, and PSP, have been lagged once.*

*GDP: Gross Domestic Product per capita. GMP: General medical practitioners per 100,000 population. EL: share (%) of the population with upper secondary education. CM: share (%) of the population having a long-standing illness or health problem (duration of at least six months). HB: hospital beds per 100,000 population. HLE: healthy life expectancy (years). NHS: National Health Service and Insurance. SHI: Social Health Insurance. ESH: Etatist Social Health Insurance. ST: State. SA: Societal actors. PA: Private actors. PGB: Prospective Global Budget. DRG: Diagnosis Related Groups. PSP: Procedure Service Payment.*

*Table S3. Results of the estimates in the WLS panel model for the period 2000-2019 excluding the number of general medical practitioners per capita.*

|  | *(1)* |  | *(2)* |  | *(3)* |  | *(4)* |  | *(5)* |  | *(6)* |  |
| --- | --- | --- | --- | --- | --- | --- | --- | --- | --- | --- | --- | --- |
| *GDP* | *-0.0410* | *** | *-0.0631* | *** | *-0.0627* | *** | *-0.0768* | *** | *-0.0789* | *** | *-0.0687* | *** |
| *GDP2* | *0.0006* | *** | *0.0009* | *** | *0.0008* | *** | *0.0009* | *** | *0.0011* | *** | *0.0010* | *** |
| *EL* | *-0.0188* | *** | *-0.0071* |  | *0.0002* |  | *0.0105* |  | *-0.0053* | *** | *-0.0130* | *** |
| *CM* | *0.0721* | *** | *0.0663* | *** | *0.0579* | *** | *0.0512* | *** | *0.0580* | *** | *0.0572* | *** |
| *HB* | *0.0060* | *** | *0.0070* | *** | *0.0066* | *** | *0.0076* | *** | *0.0052* | *** | *0.0053* | *** |
| *HLE* | *0.3993* | *** | *0.4067* | *** | *0.4023* | *** | *0.4040* | *** | *0.4192* | *** | *0.3948* | *** |
| *Time trend* | *-0.1304* | *** | *-0.1291* | *** | *-0.1330* | *** | *-0.1292* | *** | *-0.1394* | *** | *-0.1337* | *** |
| *SHI (vs NHS)* | *-0.7309* | *** |  |  |  |  |  |  |  |  | *-0.5762* | *** |
| *ESH (vs NHS)* | *0.1961* |  |  |  |  |  |  |  |  |  | *-0.0385* |  |
| *Regulation SA (vs ST)* |  |  | *-0.8356* | *** |  |  |  |  |  |  |  |  |
| *Financing SA (vs ST)* |  |  |  |  | *-0.0989* |  |  |  |  |  |  |  |
| *Provider PA (vs ST)* |  |  |  |  |  |  | *-0.6282* | *** |  |  |  |  |
| *reimbursement DRG (vs PGB)* |  |  |  |  |  |  |  |  | *1.2932* | *** | *1.2597* | *** |
| *reimbursement PSP (vs PGB)* |  |  |  |  |  |  |  |  | *1.3464* | *** | *1.1781* | *** |
| *Log-likelihood* | *-718.44* |  | *-723.04* |  | *-717.42* |  | *-719.69* |  | *-714.83* |  | *-715.36* |  |
| *AIC* | *1456.9* |  | *1464.1* |  | *1452.8* |  | *1457.4* |  | *1449.7* |  | *1454.7* |  |
| *BIC* | *1473.6* |  | *1479.1* |  | *1467.8* |  | *1472.4* |  | *1466.3* |  | *1474.7* |  |
| $R^{2}$ | *0.7828* |  | *0.7963* |  | *0.7253* |  | *0.7268* |  | *0.8250* |  | *0.8155* |  |

*Notes. * denotes a coefficient significant at 5%. All the covariates, except for the time trend and the categorical variables NHS, SHI, ESH, ST, SA, PA, PGB, DRG, and PSP, have been lagged once.*

*GDP: Gross Domestic Product per capita. GMP: General medical practitioners per 100,000 population. EL: share (%) of the population with upper secondary education. CM: share (%) of the population having a long-standing illness or health problem (duration of at least six months). HB: hospital beds per 100,000 population. HLE: healthy life expectancy (years). NHS: National Health Service and Insurance. SHI: Social Health Insurance. ESH: Etatist Social Health Insurance. ST: State. SA: Societal actors. PA: Private actors. PGB: Prospective Global Budget. DRG: Diagnosis Related Groups. PSP: Procedure Service Payment.*

*Table S4. Results of the estimates in the WLS panel model for the period 2000-2019 excluding the educational level.*

|  | *(1)* |  | *(2)* |  | *(3)* |  | *(4)* |  | *(5)* |  | *(6)* |  |
| --- | --- | --- | --- | --- | --- | --- | --- | --- | --- | --- | --- | --- |
| *GDP* | *-0.0859* | *** | *-0.0875* | *** | *-0.0933* | *** | *-0.1062* | *** | *-0.1077* | *** | *-0.1057* | *** |
| *GDP2* | *0.0010* | *** | *0.0011* | *** | *0.0011* | *** | *0.0012* | *** | *0.0014* | *** | *0.0013* | *** |
| *GMP* | *0.0091* | *** | *0.0091* | *** | *0.0103* | *** | *0.0092* | *** | *0.0079* | *** | *0.0076* | *** |
| *CM* | *0.0556* | *** | *0.0524* | *** | *0.0477* | *** | *0.0397* | *** | *0.0571* | *** | *0.0581* | *** |
| *HB* | *0.0064* | *** | *0.0060* | *** | *0.0073* | *** | *0.0078* | *** | *0.0048* | *** | *0.0051* | *** |
| *HLE* | *0.3386* | *** | *0.3359* | *** | *0.3130* | *** | *0.3593* | *** | *0.4151* | *** | *0.3925* | *** |
| *Time trend* | *-0.1222* | *** | *-0.1241* | *** | *-0.1116* | *** | *-0.1126* | *** | *-0.1428* | *** | *-0.1346* | *** |
| *SHI (vs NHS)* | *-0.81226* | *** |  |  |  |  |  |  |  |  | *-0.5420* | *** |
| *ESH (vs NHS)* | *-0.2020* |  |  |  |  |  |  |  |  |  | *-0.1113* |  |
| *Regulation SA (vs ST)* |  |  | *-0.3063* |  |  |  |  |  |  |  |  |  |
| *Financing SA (vs ST)* |  |  |  |  | *-0.6529* | *** |  |  |  |  |  |  |
| *Provider PA (vs ST)* |  |  |  |  |  |  | *-0.8771* | *** |  |  |  |  |
| *reimbursement DRG (vs PGB)* |  |  |  |  |  |  |  |  | *1.0804* | *** | *1.0886* | *** |
| *reimbursement PSP (vs PGB)* |  |  |  |  |  |  |  |  | *1.1615* | *** | *1.0794* | *** |
| *Log-likelihood* | *-718.21* |  | *-722.84* |  | *-716.77* |  | *-712.91* |  | *-714.02* |  | *-710.83* |  |
| *AIC* | *1456.4* |  | *1463.7* |  | *1451.5* |  | *1443.8* |  | *1448.0* |  | *1445.7* |  |
| *BIC* | *1473.1* |  | *1478.7* |  | *1466.5* |  | *1458.8* |  | *1464.7* |  | *1465.7* |  |
| $R^{2}$ | *0.8272* |  | *0.8304* |  | *0.8084* |  | *0.8130* |  | *0.8451* |  | *0.8717* |  |

*Notes. * denotes a coefficient significant at 5%. All the covariates, except for the time trend and the categorical variables NHS, SHI, ESH, ST, SA, PA, PGB, DRG, and PSP, have been lagged once.*

*GDP: Gross Domestic Product per capita. GMP: General medical practitioners per 100,000 population. EL: share (%) of the population with upper secondary education. CM: share (%) of the population having a long-standing illness or health problem (duration of at least six months). HB: hospital beds per 100,000 population. HLE: healthy life expectancy (years). NHS: National Health Service and Insurance. SHI: Social Health Insurance. ESH: Etatist Social Health Insurance. ST: State. SA: Societal actors. PA: Private actors. PGB: Prospective Global Budget. DRG: Diagnosis Related Groups. PSP: Procedure Service Payment.*

*Table S5. Results of the estimates in the WLS panel model for the period 2000-2019 excluding the rate of comorbidity in the population.*

|  | *(1)* |  | *(2)* |  | *(3)* |  | *(4)* |  | *(5)* |  | *(6)* |  |
| --- | --- | --- | --- | --- | --- | --- | --- | --- | --- | --- | --- | --- |
| *GDP* | *-0.1106* | *** | *-0.1048* | *** | *-0.1393* | *** | *-0.1523* | *** | *-0.1252* | *** | *-0.1218* | *** |
| *GDP2* | *0.0012* | *** | *0.0011* | *** | *0.0015* | *** | *0.0016* | *** | *0.0015* | *** | *0.0014* | *** |
| *GMP* | *0.0121* | *** | *0.0123* | *** | *0.0122* | *** | *0.0109* | *** | *0.0091* | *** | *0.0088* | *** |
| *EL* | *0.0292* | *** | *0.0271* | *** | *0.0379* | *** | *0.0402* | *** | *0.0171* | *** | *0.0162* | *** |
| *HB* | *0.0067* | *** | *0.0061* | *** | *0.0079* | *** | *0.0086* | *** | *0.0047* | *** | *0.0054* | *** |
| *HLE* | *0.3488* | *** | *0.3400* | *** | *0.3548* | *** | *0.3817* | *** | *0.4127* | *** | *0.3854* | *** |
| *Time trend* | *-0.1229* | *** | *-0.1262* | *** | *-0.1148* | *** | *-0.1165* | *** | *-0.1422* | *** | *-0.1320* | *** |
| *SHI (vs NHS)* | *-0.6194* | *** |  |  |  |  |  |  |  |  | *-0.5870* | *** |
| *ESH (vs NHS)* | *-0.2420* |  |  |  |  |  |  |  |  |  | *-0.2612* | *** |
| *Regulation SA (vs ST)* |  |  | *-0.0113* |  |  |  |  |  |  |  |  |  |
| *Financing SA (vs ST)* |  |  |  |  | *-0.9158* | *** |  |  |  |  |  |  |
| *Provider PA (vs ST)* |  |  |  |  |  |  | *-1.2467* | *** |  |  |  |  |
| *reimbursement DRG (vs PGB)* |  |  |  |  |  |  |  |  | *1.4670* | *** | *1.3814* | *** |
| *reimbursement PSP (vs PGB)* |  |  |  |  |  |  |  |  | *1.1545* | *** | *0.9943* | *** |
| *Log-likelihood* | *-713.76* |  | *-717.18* |  | *-705.74* |  | *-702.53* |  | *-712.82* |  | *-709.22* |  |
| *AIC* | *1447.5* |  | *1452.4* |  | *1429.5* |  | *1423.1* |  | *1445.8* |  | *1442.4* |  |
| *BIC* | *1464.2* |  | *1467.4* |  | *1444.5* |  | *1438.1* |  | *1462.5* |  | *1462.4* |  |
| $R^{2}$ | *0.7798* |  | *0.7838* |  | *0.7779* |  | *0.7814* |  | *0.7735* |  | *0.7865* |  |

*Notes. * denotes a coefficient significant at 5%. All the covariates, except for the time trend and the categorical variables NHS, SHI, ESH, ST, SA, PA, PGB, DRG, and PSP, have been lagged once.*

*GDP: Gross Domestic Product per capita. GMP: General medical practitioners per 100,000 population. EL: share (%) of the population with upper secondary education. CM: share (%) of the population having a long-standing illness or health problem (duration of at least six months). HB: hospital beds per 100,000 population. HLE: healthy life expectancy (years). NHS: National Health Service and Insurance. SHI: Social Health Insurance. ESH: Etatist Social Health Insurance. ST: State. SA: Societal actors. PA: Private actors. PGB: Prospective Global Budget. DRG: Diagnosis Related Groups. PSP: Procedure Service Payment.*

*Table S6. Results of the estimates in the WLS panel model for the period 2000-2019 excluding the per capita number of hospital beds.*

|  | *(1)* |  | *(2)* |  | *(3)* |  | *(4)* |  | *(5)* |  | *(6)* |  |
| --- | --- | --- | --- | --- | --- | --- | --- | --- | --- | --- | --- | --- |
| *GDP* | *-0.0149* |  | *-0.0692* | *** | *-0.0603* | *** | *-0.0329* | *** | *0.0143* |  | *-0.0249* | *** |
| *GDP2* | *0.0003* | *** | *0.0007* | *** | *0.0007* | *** | *0.0006* | *** | *0.0002* | *** | *0.0006* | *** |
| *GMP* | *0.0080* | *** | *0.0071* | *** | *0.0071* | *** | *0.0086* | *** | *0.0066* | *** | *0.0086* | *** |
| *EL* | *-0.0672* | *** | *-0.0237* | *** | *-0.0462* | *** | *-0.0569* | *** | *-0.0326* | *** | *-0.0434* | *** |
| *CM* | *0.0755* | *** | *0.0623* | *** | *0.0680* | *** | *0.0898* | *** | *0.0614* | *** | *0.0654* | *** |
| *HLE* | *0.3203* | *** | *0.1151* | *** | *0.3289* | *** | *0.2050* | *** | *0.0039* | *** | *0.2264* | *** |
| *Time trend* | *-0.1339* | *** | *-0.1179* | *** | *-0.1481* | *** | *-0.1299* | *** | *-0.0859* | *** | *-0.1278* | *** |
| *SHI (vs NHS)* | *0.3912* | *** |  |  |  |  |  |  |  |  | *-0.1619* |  |
| *ESH (vs NHS)* | *1.6383* | *** |  |  |  |  |  |  |  |  | *1.1599* | *** |
| *Regulation SA (vs ST)* |  |  | *1.6743* | *** |  |  |  |  |  |  |  |  |
| *Financing SA (vs ST)* |  |  |  |  | *1.1374* | *** |  |  |  |  |  |  |
| *Provider PA (vs ST)* |  |  |  |  |  |  | *1.0566* | *** |  |  |  |  |
| *reimbursement DRG (vs PGB)* |  |  |  |  |  |  |  |  | *1.2469* | *** | *1.8553* | *** |
| *reimbursement PSP (vs PGB)* |  |  |  |  |  |  |  |  | *1.0427* | *** | *1.3971* | *** |
| *Log-likelihood* | *-702.21* |  | *-709.67* |  | *-700.03* |  | *-716.80* |  | *-756.91* |  | *-713.25* |  |
| *AIC* | *1424.4* |  | *1437.3* |  | *1418.1* |  | *1451.6* |  | *1533.8* |  | *1450.5* |  |
| *BIC* | *1441.1* |  | *1452.3* |  | *1433.1* |  | *1466.6* |  | *1550.6* |  | *1470.5* |  |
| $R^{2}$ | *0.7064* |  | *0.5066* |  | *0.5688* |  | *0.5650* |  | *0.8000* |  | *0.8526* |  |

*Notes. * denotes a coefficient significant at 5%. All the covariates, except for the time trend and the categorical variables NHS, SHI, ESH, ST, SA, PA, PGB, DRG, and PSP, have been lagged once.*

*GDP: Gross Domestic Product per capita. GMP: General medical practitioners per 100,000 population. EL: share (%) of the population with upper secondary education. CM: share (%) of the population having a long-standing illness or health problem (duration of at least six months). HB: hospital beds per 100,000 population. HLE: healthy life expectancy (years). NHS: National Health Service and Insurance. SHI: Social Health Insurance. ESH: Etatist Social Health Insurance. ST: State. SA: Societal actors. PA: Private actors. PGB: Prospective Global Budget. DRG: Diagnosis Related Groups. PSP: Procedure Service Payment.*

*Table S7. Results of the estimates in the WLS panel model for the period 2000-2019 excluding the healthy life expectancy.*

|  | *(1)* |  | *(2)* |  | *(3)* |  | *(4)* |  | *(5)* |  | *(6)* |  |
| --- | --- | --- | --- | --- | --- | --- | --- | --- | --- | --- | --- | --- |
| *GDP* | *0.0137* |  | *0.0013* |  | *-0.0134* |  | *-0.0207* |  | *0.0143* |  | *0.0149* |  |
| *GDP2* | *0.0001* |  | *0.0002* |  | *0.0004* | *** | *0.0004* | *** | *0.0002* | *** | *0.0003* | *** |
| *GMP* | *0.0090* | *** | *0.0092* | *** | *0.0097* | *** | *0.0088* | *** | *0.0065* | *** | *0.0065* | *** |
| *EL* | *-0.0417* | *** | *-0.0333* | *** | *-0.0183* | *** | *-0.0186* | *** | *-0.0326* | *** | *-0.0359* | *** |
| *CM* | *0.0646* | *** | *0.0537* | *** | *0.0493* | *** | *0.0402* | *** | *0.0614* | *** | *0.0650* | *** |
| *HB* | *0.0056* | *** | *0.0052* | *** | *0.0062* | *** | *0.0062* | *** | *0.0039* | *** | *0.0042* | *** |
| *Time trend* | *-0.1275* | *** | *-0.0709* | *** | *-0.0681* | *** | *-0.0672* | *** | *-0.0859* | *** | *-0.0822* | *** |
| *SHI (vs NHS)* | *-0.7433* | *** |  |  |  |  |  |  |  |  | *-0.6826* | *** |
| *ESH (vs NHS)* | *-0.1275* |  |  |  |  |  |  |  |  |  | *-0.0821* |  |
| *Regulation SA (vs ST)* |  |  | *0.1145* |  |  |  |  |  |  |  |  |  |
| *Financing SA (vs ST)* |  |  |  |  | *-0.5172* | *** |  |  |  |  |  |  |
| *Provider PA (vs ST)* |  |  |  |  |  |  | *-0.6130* | *** |  |  |  |  |
| *reimbursement DRG (vs PGB)* |  |  |  |  |  |  |  |  | *1.2469* | *** | *1.2584* | *** |
| *reimbursement PSP (vs PGB)* |  |  |  |  |  |  |  |  | *1.0427* | *** | *0.9689* | *** |
| *Log-likelihood* | *-752.53* |  | *-751.10* |  | *-754.32* |  | *-756.02* |  | *-756.91* |  | *-757.61* |  |
| *AIC* | *1525.1* |  | *1520.2* |  | *1526.6* |  | *1530.0* |  | *1533.8* |  | *1539.2* |  |
| *BIC* | *1541.9* |  | *1535.3* |  | *1541.8* |  | *1545.2* |  | *1550.6* |  | *1559.4* |  |
| $R^{2}$ | *0.7234* |  | *0.7640* |  | *0.8112* |  | *0.8159* |  | *0.8000* |  | *0.8410* |  |

*Notes. * denotes a coefficient significant at 5%. All the covariates, except for the time trend and the categorical variables NHS, SHI, ESH, ST, SA, PA, PGB, DRG, and PSP, have been lagged once.*

*GDP: Gross Domestic Product per capita. GMP: General medical practitioners per 100,000 population. EL: share (%) of the population with upper secondary education. CM: share (%) of the population having a long-standing illness or health problem (duration of at least six months). HB: hospital beds per 100,000 population. HLE: healthy life expectancy (years). NHS: National Health Service and Insurance. SHI: Social Health Insurance. ESH: Etatist Social Health Insurance. ST: State. SA: Societal actors. PA: Private actors. PGB: Prospective Global Budget. DRG: Diagnosis Related Groups. PSP: Procedure Service Payment.*

*Table S8. Results of the estimates in the WLS panel model for the period 2000-2019 excluding Finland and Turkey.*

|  | *(1)* |  | *(2)* |  | *(3)* |  | *(4)* |  | *(5)* |  | *(6)* |  |
| --- | --- | --- | --- | --- | --- | --- | --- | --- | --- | --- | --- | --- |
| *GDP* | *-0.0704* | *** | *-0.1025* | *** | *-0.1036* | *** | *-0.0808* | *** | *-0.1060* | *** | *-0.0865* | *** |
| *GDP2* | *0.0009* | *** | *0.0011* | *** | *0.0013* | *** | *0.0011* | *** | *0.0013* | *** | *0.0011* | *** |
| *GMP* | *0.0066* | *** | *0.0075* | *** | *0.0070* | *** | *0.0059* | *** | *0.0068* | *** | *0.0074* | *** |
| *EL* | *-0.0437* | *** | *-0.0196* | *** | *-0.0254* | *** | *-0.0477* | *** | *-0.0135* | *** | *-0.0339* | *** |
| *CM* | *0.0400* | *** | *0.0397* | *** | *0.0500* | *** | *0.0494* | *** | *0.0425* | *** | *0.0435* | *** |
| *HB* | *0.0025* | *** | *0.0046* | *** | *0.0055* | *** | *0.0035* | *** | *0.0044* | *** | *0.0027* | *** |
| *HLE* | *0.3439* | *** | *0.3407* | *** | *0.3893* | *** | *0.3905* | *** | *0.3820* | *** | *0.3701* | *** |
| *Time trend* | *-0.1188* | *** | *-0.1160* | *** | *-0.1191* | *** | *-0.1187* | *** | *-0.1236* | *** | *-0.1231* | *** |
| *SHI (vs NHS)* | *-0.3094* | *** |  |  |  |  |  |  |  |  | *-0.6535* | *** |
| *ESH (vs NHS)* | *0.4778* | *** |  |  |  |  |  |  |  |  | *0.7522* | *** |
| *Regulation SA (vs ST)* |  |  | *0.2529* |  |  |  |  |  |  |  |  |  |
| *Financing SA (vs ST)* |  |  |  |  | *-0.5634* | *** |  |  |  |  |  |  |
| *Provider PA (vs ST)* |  |  |  |  |  |  | *-0.9685* | *** |  |  |  |  |
| *reimbursement DRG (vs PGB)* |  |  |  |  |  |  |  |  | *0.9603* | *** | *0.8149* | *** |
| *reimbursement PSP (vs PGB)* |  |  |  |  |  |  |  |  | *1.0132* | *** | *0.8521* | *** |
| *Log-likelihood* | *-693.59* |  | *–664.21* |  | *-660.48* |  | *-667.94* |  | *-659.10* |  | *-657.83* |  |
| *AIC* | *1409.2* |  | *1348.4* |  | *1340.9* |  | *1355.9* |  | *1340.2* |  | *1341.7* |  |
| *BIC* | *1427.4* |  | *1364.8* |  | *1357.4* |  | *1372.4* |  | *1358.3* |  | *1362.9* |  |
| $R^{2}$ | *0.8052* |  | *0.7056* |  | *0.7257* |  | *0.8721* |  | *0.8318* |  | *0.8321* |  |

*Notes. * denotes a coefficient significant at 5%. All the covariates, except for the time trend and the categorical variables NHS, SHI, ESH, ST, SA, PA, PGB, DRG, and PSP, have been lagged once.*

*GDP: Gross Domestic Product per capita. GMP: General medical practitioners per 100,000 population. EL: share (%) of the population with upper secondary education. CM: share (%) of the population having a long-standing illness or health problem (duration of at least six months). HB: hospital beds per 100,000 population. HLE: healthy life expectancy (years). NHS: National Health Service and Insurance. SHI: Social Health Insurance. ESH: Etatist Social Health Insurance. ST: State. SA: Societal actors. PA: Private actors. PGB: Prospective Global Budget. DRG: Diagnosis Related Groups. PSP: Procedure Service Payment.*
